# Coexpression Pattern Analysis of NPM1-Associated Genes in Chronic Myelogenous Leukemia

**DOI:** 10.1155/2015/610595

**Published:** 2015-04-15

**Authors:** Fengfeng Wang, Lawrence W. C. Chan, Nancy B. Y. Tsui, S. C. Cesar Wong, Parco M. Siu, S. P. Yip, Benjamin Y. M. Yung

**Affiliations:** Department of Health Technology and Informatics, Hong Kong Polytechnic University, Hung Hom, Kowloon, Hong Kong

## Abstract

*Background*. Nucleophosmin 1 (NPM1) plays an important role in ribosomal synthesis and malignancies, but NPM1 mutations occur rarely in the blast-crisis and chronic-phase chronic myelogenous leukemia (CML) patients. The NPM1-associated gene set (GCM_NPM1), in total 116 genes including NPM1, was chosen as the candidate gene set for the coexpression analysis. We wonder if NPM1-associated genes can affect the ribosomal synthesis and translation process in CML. *Results*. We presented a distribution-based approach for gene pair classification by identifying a disease-specific cutoff point that classified the coexpressed gene pairs into strong and weak coexpression structures. The differences in the coexpression patterns between the normal and the CML groups were reflected from the overall structure by performing two-sample Kolmogorov-Smirnov test. Our developed method effectively identified the coexpression pattern differences from the overall structure: *P*  value = 1.71 × 10^−22^ < 0.05 for the maximum deviation *D* = 0.109. Moreover, we found that genes involved in the ribosomal synthesis and translation process tended to be coexpressed in the CML group. *Conclusion*. Our developed method can identify the coexpression difference between two different groups. Dysregulation of ribosomal synthesis and translation process may be related to the CML disease. Our significant findings may provide useful information for the novel CML mechanism exploration and cancer treatment.

## 1. Introduction

Nucleophosmin 1 (NPM1), also named nucleolar phosphoprotein B23, belongs to the NucleoPhosMin/NucleoPlasMin family of nuclear chaperones. The whole family can be divided into four classes based on protein sequence similarities: nucleophosmin (NPM1), nucleoplasmin 2 (NPM2), nucleoplasmin 3 (NPM3), and NPM-like invertebrate proteins [1, 2]. NPM1 is well studied in the whole family with its cDNA cloned in 1989, encoding a 294-amino-acid protein [3]. The expression of NPM1 gene is frequently altered in solid tumors, and its mutation and translocation are also found in hematological malignancies [4]. The encoded protein product is a phosphoprotein that travels between the nucleus and cytoplasm, which plays multiple roles in ribosomal RNA (rRNA) processing, ribosome assembly, transport of ribosomal subunits, centrosome duplication, regulation of p53, and cell growth and proliferation [5–7].

According to the gene list curated by Brentani et al., NPM1 is one of 380 cancer-associated genes obtained from a published cancer gene database [8]. In a study of Subramanian et al., the neighborhoods highly correlated with these cancer-associated genes were selected based on four large gene expression datasets that were collected from various cancer projects mainly on primary tumors, including prostate, breast, lung, lymphoma, and leukemia [9]. Pearson correlation coefficient (*r*) between every gene in these four datasets and one cancer-associated gene (e.g., NPM1) was calculated independently in each dataset. A gene was selected as the neighborhood if *r* ≥ 0.85 in at least one out of four datasets. The cancer-associated genes with no less than 25 selected neighborhoods were stored in the* Molecular Signature Database* (*MSigDB*) [9]. The NPM1-associated gene set (GCM_NPM1), in total 116 genes including NPM1, is one of the neighborhood sets [9].

NPM1 often participates in chromosomal translocation, mutation, and deletion in hematological malignancies [5]. Chronic myelogenous leukemia (CML) is a clonal myeloproliferative disorder, which is characterized by the increased and unregulated growth of immature myeloid cells in the blood stream [10]. The cytoplasmic mutated NPM1 was found for the first time in a blast-crisis CML patient, indicating that the mutation of NPM1 gene may function in the blastic transformation of CML [11]. Interestingly, in a recent study, researchers did not detect any NPM1 mutations in the analyzed blast-crisis and chronic-phase CML patients [12]. We wonder if NPM1-associated genes can affect the ribosomal synthesis and translation process in CML. Coexpression analysis has been applied to study the functionally related genes, since the coexpressed genes are more likely to participate in the similar biological processes and signaling pathways [13, 14].

In this study, we aim to explore the differences in the coexpression patterns of those NPM1-associated genes between the normal and the CML states, to further investigate the altered ribosome activities in CML. We proposed a method to explore the coexpression pattern difference by identifying a disease-specific cutoff point that classified the coexpressed gene pairs into strong and weak coexpression classes so that the class was best coherent with the CML state. Traditional methods on the gene coexpression analysis calculate the individual *P* value of correlation coefficient for every gene pair to identify the significantly coexpressed gene pairs. Our developed method considered the correlation coefficients for all the gene pairs in each group to form two different cumulative distributions, which can identify the difference between two different groups from the overall structure. The different coexpression pattern indicated the biological alterations in CML. In addition, the functional annotation of coexpressed gene pairs provided useful information to understand the underlying mechanisms of the CML disease.

## 2. Methods

### 2.1. Microarray Expression Data

Microarray technology is useful to extract the important information from cells. Different conditions have different gene expression levels. In this study, we chose the microarray dataset GSE5550 normalized by variance stabilizing transformations (VSN) method, which is publicly available on the* Gene Expression Omnibus* (*GEO*) repository [15]. The data in this dataset are obtained from gene expression measurements of more than 8,000 unique mRNAs. CD34+ hematopoietic stem and progenitor cells were collected from the bone marrows of untreated CML patients in the chronic phase and healthy controls [15]. The subjects recruited for this dataset are Caucasians from Germany. Two groups are included in this dataset: (i) the CML group, nine patients, and (ii) the control group, eight normal subjects. In this dataset, a gene may be interrogated by more than one probe. We took the average of all the probes for the same mRNA to deal with this situation [16, 17].

### 2.2. Coexpression Measure

There were 93 out of 116 NPM1-associated genes found in the CML microarray dataset GSE5550 (see Table  S1 in Supplementary Material available online at http://dx.doi.org/10.1155/2015/610595). We extracted the expression profiles of these 93 genes for the coexpression analysis. The expression matrix was in dimension of 93 × 17, where each row referred to the relative expression levels of a gene across all the samples (8 normal and 9 CML samples). In this study, Pearson correlation coefficient (*r*) was chosen as the similarity measure to indicate the associations between genes [18]. Pearson correlation coefficient can be used to demonstrate the biological relationship of two genes numerically, which does not emphasize the magnitude of their expression profiles [13, 19]. The similarity measure is usually regarded as a kernel function between two feature vectors.

In this study, each feature vector included the expression profiles of a gene across all the samples in the normal group or the CML group, respectively. The absolute values of correlation coefficients (|*r*| values) were chosen, since the coexpression measure output a scalar in the range from 0 to 1 where a high value demonstrated a strong biological relationship in either positive or negative direction and a low value indicated a weak biological relationship. *C*_*d*_(*i*, *j*) referred to the coexpression level of two genes from the disease (CML) group, and *C*_*n*_(*i*, *j*) was for the normal group (Formulas 1) [18]:

(1) Cdi,jcorxdi,xdj,Cni,j=corxni,xnj,

where *C*_*d*_(*i*, *j*) and *C*_*n*_(*i*, *j*) are the absolute values of correlation coefficients for genes *i* and *j* in the CML group and the normal group, respectively [19]; *x*_*di*_ and *x*_*dj*_ refer to the expression profiles of the *i*th and *j*th genes in the CML group; *x*_*ni*_ and *x*_*nj*_ refer to the expression profiles of the *i*th and *j*th genes in the normal group; cor(*x*_*di*_, *x*_*dj*_) and cor(*x*_*ni*_, *x*_*nj*_) are the Pearson correlation coefficients in the CML group and the normal group, respectively.

### 2.3. Identification of the Disease-Specific Cutoff Point for Gene Pair Classification

Two sets of correlation coefficients in the normal and the CML groups formed two different cumulative distributions. Two-sample Kolmogorov-Smirnov (KS) test was applied to test if these two sets of data significantly differed in terms of the overall distributions. The significance for KS test was indicated by the *P* value for the maximum deviation between two cumulative distributions of *C*_*d*_ and *C*_*n*_ (Formulas 2). At the maximum deviation, a threshold was identified to group the coexpressed gene pairs into strong and weak coexpression classes, called the disease-specific cutoff point (*C*). The cutoff point represented a coexpression level, at which *F*_*d*_ and *F*_*n*_ were extremely deviated:

(2) D=max⁡CFdC−FnC,FdC=ProbCd≥C,FnC=ProbCn≥C,

where *F*_*d*_ and *F*_*n*_ refer to the cumulative distribution functions of *C*_*d*_ and *C*_*n*_, respectively; *D* represents the maximum deviation; *C* is the cutoff point.

The specifically coexpressed gene pairs were further identified in different groups. Different types of gene pairs indicated different biological meanings. The normal-specific strongly coexpressed pairs included the gene pairs strongly coexpressed only in the normal group, which represented the physiological balance in the cells of healthy individuals. Apparently, these pairs were the CML-specific weakly coexpressed pairs that were weakly coexpressed only in the CML group. The CML-specific strongly coexpressed pairs included the gene pairs strongly coexpressed only in the CML group, which demonstrated the characteristics of the disease. For the same reason, these pairs were the normal-specific weakly coexpressed pairs.

### 2.4. Functional Annotation for NPM1-Associated Genes Using* DAVID* Database

Gene ontology (GO) provides a systematic language or ontology to describe gene and gene product attributes across all species [20]. It can be classified into three categories [21]: (i) biological process: a set of molecular events with a defined beginning and end, for example, a chemical or physical transformation; (ii) cellular component: the parts of a cell or the extracellular environment where a gene product is active; and (iii) molecular function: the elemental activities of a gene product at the molecular level, for example, the specific binding to ligands and catalysis. We applied gene ontology to group the NPM1-associated genes into different classes, to further explore the biological meaning of the coexpressed gene pairs in the CML state.

The* Database for Annotation, Visualization and Integrated Discovery* (*DAVID*) was chosen to annotate these 93 genes, which is useful to extract the biological meaning by combining an integrated biological knowledge base and multiple analytic tools [22]. All these three GO categories (biological process, molecular function, and cellular component) were considered in our study. Functional annotation chart was used to identify the significant batch annotation and GO terms that were most pertinent to the input data. When the NPM1-associated gene list was uploaded to* DAVID*, the annotation chart provided the significantly enriched GO terms. The significance of GO term enrichment is calculated according to a modified Fisher exact test, Expression Analysis Systematic Explorer (EASE) score. The EASE score is regarded as a more conservative and robust adjustment than the Fisher exact probability [23].* DAVID* also provides false discovery rate (FDR) to control the expected proportion of false positives for the multiple hypotheses. The selection criteria for the significantly enriched GO terms used in our study were (i) EASE score < 0.05 and (ii) FDR < 0.05.

### 2.5. Mapping Coexpressed Gene Pairs to Annotated Gene Pairs

The annotated genes in each enriched GO term were paired with all the possible combinations, forming the annotated gene pairs. The annotated gene pairs were mapped to the identified coexpressed gene pairs in each GO term: the mapped CML-specific strongly coexpressed, the mapped CML-specific weakly coexpressed, the mapped normal-specific strongly coexpressed, and the mapped normal-specific weakly coexpressed pairs. Fisher exact test was used to verify if genes were more likely to be coexpressed in the CML group compared to the normal group. As a result, one-sided *P* value was chosen to indicate the significance. The multiple-hypothesis test was performed on a list of mapped GO terms by applying the more stringent Bonferroni correction. The *P* value of Fisher exact test was multiplied by the total number of considered GO terms. A GO term was significantly mapped if its corrected *P* value was still smaller than 0.05.

## 3. Results

### 3.1. Identification of Structural Coexpression Difference

**Figure 1:**
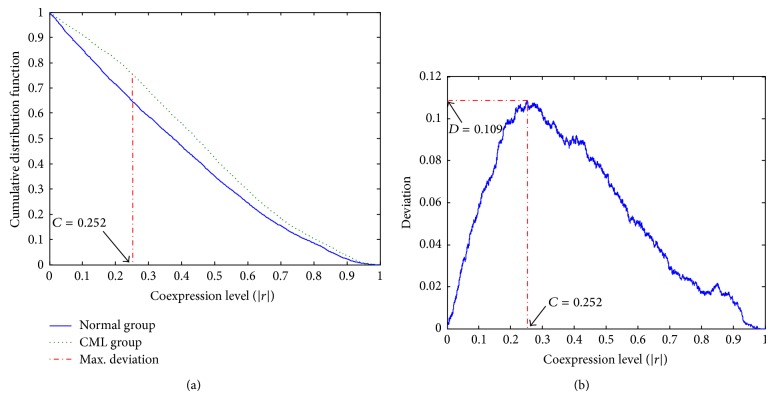
Plots of distributions for the 93 NPM1-associated genes coexpression analysis. (a) Cumulative distribution functions of coexpression levels in the normal and the CML groups. (b) Deviation distribution against different coexpression cutoff points.

The correlation coefficients for all the possible gene pair combinations of these 93 NPM1-associated genes were calculated. In each group, there was a set of correlation coefficients of 4,278 gene pairs. The cumulative distributions of these two sets of data were plotted (Figure 1). The results for KS test showed that the two distributions in the normal and the CML groups were significantly different from the overall structure (*P* value = 1.71 × 10^−22^ < 0.05 for the maximum deviation *D* = 0.109).

The disease-specific cutoff point, *C* = 0.252, was identified at the maximum deviation (Figure 1). Two coexpression patterns were so distinct that the CML group had more strongly coexpressed (level above ~0.252) and less weakly coexpressed (level below ~0.252) gene pairs than that in the normal group. The cutoff point classified gene pairs into four coexpression classes (Table 1). Binomial distribution test indicated that the proportion of strongly coexpressed gene pairs in the CML group was significantly higher than that in the normal group (one-sided *P* value <0.001).

### 3.2. *DAVID* Database Annotation for Enriched Biological Process

According to the selection criteria (EASE score < 0.05 and FDR < 0.05), eight significantly enriched GO terms for biological processes were identified (see Table S2). We obtained the annotated genes involved in each biological process and formed the annotated gene pairs. Then, the coexpressed gene pairs were mapped to the annotated gene pairs. The results showed that all these eight processes had more mapped CML-specific strongly coexpressed pairs (Table 2). In other words, genes were more likely to be coexpressed in the CML group when compared to the normal group. Fisher exact test was used to indicate the significance. The results showed that* translational elongation*,* translation*,* cellular protein metabolic process*,* RNA processing*, and* RNA metabolic process* were significantly mapped (*P* values <0.05 and corrected *P* values <0.05).

**Figure 2:**
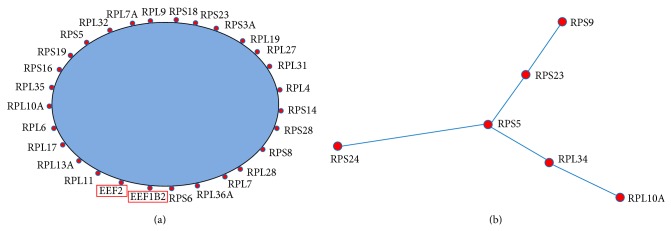
Simplified coexpression networks for the mapped strongly coexpressed pairs in the* translational elongation* biological process (see Figure S1 for the detailed networks). The blue area is for the omitted connections among genes. Genes with red rectangles are not RP genes. (a) Mapped CML-specific strongly coexpressed pairs. (b) Mapped normal-specific strongly coexpressed pairs.

**Figure 3:**
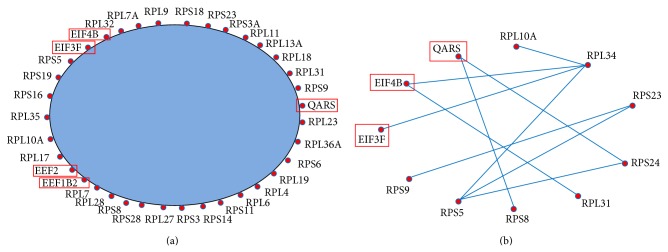
Simplified coexpression networks for the mapped strongly coexpressed pairs in the* translation* biological process (see Figure S2 for the detailed networks). The blue area is for the omitted connections among genes. Genes with red rectangles are not RP genes. (a) Mapped CML-specific strongly coexpressed pairs. (b) Mapped normal-specific strongly coexpressed pairs.

*Translational elongation* and* translation* were related to gene translation process.* Translational elongation* is defined as the successive addition of amino acid residues to a nascent polypeptide chain in the protein biosynthesis process.* Translation* refers to the cellular metabolic process to form a protein by using a mature mRNA molecule to determine the amino acids sequence in a polypeptide chain. We further plotted the coexpression networks for the strongly coexpressed gene pairs in the normal and the CML groups (Figures 2, 3, S1, and S2). From the coexpression networks, we also observed that there were more connections in the CML group compared to the normal group (Figures S1 and S2). Genes identified in the coexpression networks were classified into two major classes: (i) ribosomal protein (RP) genes, such as ribosomal protein L6 (RPL6) and ribosomal protein S28 (RPS28), and (ii) translation factors, such as eukaryotic translation elongation factor 2 (EEF2) and eukaryotic translation initiation factor 3, subunit F (EIF3F). The results revealed that nearly all the coexpressed genes were RP genes, which are responsible for encoding the ribosomal small and large subunits.

The basic information for the identified translation factors was obtained from* National Center for Biotechnology Information *(*NCBI*) database. Protein products from EEF2 and EEF1B2 belong to translation elongation factors. EEF2 is a member of the GTP-binding translation elongation factor family, which is very important for protein synthesis. This protein can mediate the process of GTP-dependent translocation of the nascent protein chain from A-site to P-site on the ribosome. The encoded protein of EEF1B2 is a guanine nucleotide exchange factor responsible for the transfer of aminoacylated transfer RNAs (tRNAs) to the ribosome. Eukaryotic translation initiation factor 3, subunit F, and initiation factor 4B (EIF3F and EIF4B) are translation initiation factors, which are vital to initiate the translation.

### 3.3. *DAVID* Database Annotation for Enriched Cellular Component

Based on the same selection criteria (EASE score < 0.05 and FDR < 0.05), 21 significantly enriched GO terms for cellular components were identified (see Table S3). The annotated genes involved in each GO term were obtained and formed the annotated gene pairs. We also mapped the coexpressed gene pairs to the annotated gene pairs. The results demonstrated that genes were more likely to be coexpressed in the CML group when compared to the normal group among 18 out of 21 GO terms (Table 3). Fisher exact test showed that* ribonucleoprotein complex*,* ribosome*,* cytosolic ribosome*,* ribosomal subunit*,* cytosol*,* cytosolic part*,* intracellular non-membrane-bounded organelle*,* intracellular organelle part*,* cytosolic large ribosomal subunit*,* cytoplasmic part*,* cytoplasm*,* intracellular organelle*,* nuclear part*,* nuclear lumen*,* intracellular organelle lumen*, and* nucleolus* were significantly mapped (*P* values <0.05, and corrected *P* values <0.05).

In these significantly mapped GO terms, five of them were related to ribosome:* ribonucleoprotein complex*,* ribosome*,* cytosolic ribosome*,* ribosomal subunit*, and* cytosolic large ribosomal subunit*.* Ribonucleoprotein complex* refers to a macromolecular complex consisting of both proteins and RNA molecules.* Ribosome* contains large and small subunits, as well as other proteins and RNAs, which is regarded as a machine for protein biosynthesis.* Cytosolic ribosome* describes a ribosome that is located in the cytosol.* Ribosomal subunit* consists of ribosomal large and small subunits.* Cytosolic large ribosomal subunit* refers to the large subunit that is located in the cytosol. There were more connections in the CML group compared to the normal group (Table 3). In addition, most of the coexpressed genes belonged to RP genes encoding the ribosomal large and small subunits.

**Figure 4:**
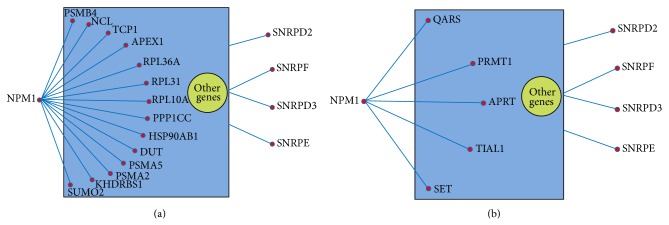
Simplified coexpression networks for the mapped strongly coexpressed pairs in the* cytoplasm* cellular component (see Figure  for the detailed networks). Genes coexpressed with NPM1 and genes encoding small nuclear ribonucleoproteins are shown in the networks. The other genes and the omitted connections among genes are demonstrated in the yellow and blue areas. (a) Mapped CML-specific strongly coexpressed pairs. (b) Mapped normal-specific strongly coexpressed pairs.

**Figure 5:**
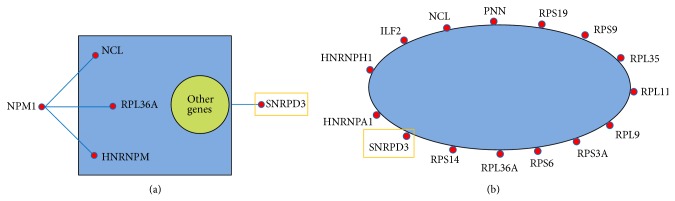
Simplified coexpression networks for the mapped strongly coexpressed pairs in the* nucleolus* cellular component (see Figure S4 for the detailed networks). Genes with yellow rectangles refer to those genes encoding small nuclear ribonucleoproteins. The other genes and the omitted connections among genes are demonstrated in the yellow and blue areas. (a) Mapped CML-specific strongly coexpressed pairs. Genes coexpressed with NPM1 are shown in the network. (b) Mapped normal-specific strongly coexpressed pairs.

The nucleolus is very important for ribosome biogenesis, containing the proteins for ribosome production [24, 25]. A number of nucleoli are found to be centered around rDNAs that are transcribed to rRNAs for ribosome [25, 26]. In addition, various proteins responsible for the processing and assembly of ribosomal large and small subunits are also included in the nucleolus [25]. We found that genes encoding small nuclear ribonucleoproteins were well connected with other genes in the CML group: small nuclear ribonucleoprotein D2 polypeptide 16.5 kDa (SNRPD2), D3 polypeptide 18 kDa (SNRPD3), polypeptide E (SNRPE), and polypeptide F (SNRPF) (Figures 4 and 5). From the figures, we can see that these small nuclear ribonucleoprotein genes had more connections with other genes in the CML networks compared to the normal networks (Figures S3 and S4). It was reported that NPM1 can shuttle from the nucleus to the cytoplasm [27]. NPM1 was also found to direct the nuclear export of ribosome [25]. When exported to the cytoplasm, the small and large subunits are combined together to form functional subunits [25]. In our result, NPM1 was found in both* cytoplasm* and* nucleolus* GO terms for cellular components. Most importantly, NPM1 was coexpressed with more genes in the CML group than that in the normal group, including the RP genes, for example, RPL10A and RPL36A (Figures 4 and 5).

There was no significantly enriched GO term for molecular function identified according to the same selection criteria (EASE score < 0.05 and FDR < 0.05).

## 4. Discussion and Conclusion

In this study, we have identified the overall differences in the coexpression patterns of those NPM1-associated genes between the normal and the CML groups. Correlation coefficients for all the possible gene pairs among these 93 genes were considered to form two different cumulative distributions. Two-sample KS test was performed to identify the difference (Figure 1). Firstly, the maximum deviation (*D* = 0.109) between two cumulative distributions indicated the difference between the normal and the CML groups structurally. Then, a disease-specific cutoff point (*C* = 0.252) was discovered at the maximum deviation to classify the coexpressed gene pairs. Functional annotation was further applied to explore the biological differences.

*DAVID *database annotation for enriched biological process gene ontology demonstrated that genes involved in* translational elongation* and* translation* were more likely to be coexpressed in the CML group, which were related to translation process (Table 2). The coexpressed genes that participated in these two biological processes covered RP genes (e.g., RPL6 and RPS28) and translation factors (e.g., EEF2 and EIF3F) (Figures 2 and 3). The RP genes are responsible for encoding the ribosomal large and small subunits. Ribosome is regarded as a machine for protein biosynthesis. There are some factors needed to assist the translation process, including initiation factors and elongation factors. In the significantly mapped GO terms for cellular components, some of them were related to ribosome, cytoplasm, and nucleolus (Table 3 and Figures 4 and 5). The rRNA large and small subunits are generated in the nucleolus. After exported to the cytoplasm, these components are combined together to form the functional ribosome to perform the translation function. Therefore, both the biological processes and the cellular components are important. Our results showed that genes involved in the translation processes, ribosome, cytoplasm, and nucleolus were more likely to be coexpressed in the CML group compared to the normal group. We can infer that the ribosome biogenesis and translation process may be more active in the CML state.

The translation process, ribosomal protein, and translation factor have been found dysregulated in the CML state. Altered mRNA translation is involved in the pathogenesis of various human cancers, including CML [28]. Ly et al. reported that the translational regulators, ribosomal protein S6 and 4E-BP1 (a negative regulator in cap-dependent mRNA translation process), were constitutively phosphorylated in CML cells [29]. The encoded protein by eukaryotic translation initiation factor 4E (EIF4E) is regarded as both a key translation factor and a promoter for nucleocytoplasmic transport of specific transcripts [30]. Overexpression of EIF4E has been found in CML patients, suggesting its possible role in neoplastic transformation and the feasibility as a novel therapeutic approach [30, 31].

Our developed method had two major functions. Two sets of correlation coefficients in the normal and the CML groups formed two different cumulative distributions. The first function was to test if these two sets of data significantly differed in terms of the overall distributions. The significance was indicated by the *P* value for the maximum deviation between two cumulative distributions. A threshold was identified at the maximum deviation to group the coexpressed gene pairs into strong and weak coexpression classes, called the disease-specific cutoff point, which was regarded as the second function. The widely appreciated cutoffs for the *P* values such as 0.01 or 0.05 can identify the strong and weak coexpression classes pair by pair. However, it cannot test the difference from the overall distributions of two groups.

Gene differential expression analysis applies statistical methods to select genes with high/low expression levels in the disease group and low/high expression levels in the normal state [32]. Individual gene expression value change is able to indicate the possible relation between this gene and disease but cannot identify the interaction between different genes and the plurality of pathogenic genes as a functional module in the complex disease [33, 34]. In the real situation, genes and their encoded proteins do not function in isolation, and they cooperate with each other [35, 36]. Functional changes such as the alteration in a particular biological process can be reflected by gene coexpression changes [34]. Compared to the gene differential expression analysis, coexpression analysis is able to identify the functional relationship among genes during signal transduction and group genes involved in a functional gene set or a particular pathway. Hence, the coexpression analysis is more useful for analyzing the underlying mechanisms of diseases. The altered coexpression patterns in the CML state with respect to the normal state can be used to identify the dysregulated pathways more easily.

We have developed a novel method to identify a disease-specific cutoff point for coexpression levels that classified the coexpressed gene pairs. This distribution-based classification considered all the gene pairs to partition them into different locations based on their different coexpression levels and different groups. We applied this method to explore the difference in the coexpression patterns of those NPM1-associated genes between the normal and the CML groups. Our method effectively identified the statistical differences from the overall structure. The different coexpression pattern compared to the normal state reflected the biological alterations in CML. Moreover, dysregulated ribosomal synthesis and translation process were found in the CML state compared to the normal group. Our developed method and significant findings may provide useful information for the exploration of novel mechanisms and the treatment of cancer.

## Supplementary Material

The detailed coexpression networks for the mapped strongly coexpressed pairs are shown in Figures S1, S2, S3 and S4. The 93 NPM1-associated genes that can be found in the microarray dataset GSE5550 are shown in Table S1. The enriched biological process GO terms for the functional annotation of NPM1-associated genes are shown in Table S2. The enriched cellular component GO terms for the functional annotation of NPM1-associated genes are shown in Table S3.

## Figures and Tables

**Table 1:** The coexpressed gene pairs identified by the disease-specific cutoff point.

Group	Number of strongly coexpressed gene pairs	Number of weakly coexpressed gene pairs

Normal	2763	1515
CML	3228	1050

**Table 2:** Mapping coexpressed gene pairs to annotated gene pairs from each biological process.

Number	GO terms	Fisher exact test					Corrected *P* value
		*a *	*b *	*c *	*d *	*P* value	
1	**Translational elongation**	**59**	**5**	**5**	**59**	***<0.001***	***<0.008***
2	**Translation**	**89**	**10**	**10**	**89**	***<0.001***	***<0.008***
3	**Cellular protein metabolic process**	**299**	**116**	**116**	**299**	***<0.001***	***<0.008***
4	**RNA processing**	**63**	**28**	**28**	**63**	***<0.001***	***<0.008***
5	**RNA metabolic process**	**84**	**39**	**39**	**84**	***<0.001***	***<0.008***
6	mRNA processing	18	11	11	18	0.057	0.456
7	RNA splicing	16	10	10	16	0.082	0.656
8	mRNA metabolic process	18	11	11	18	0.057	0.456

GO: gene ontology. GO terms highlighted in bold text are significantly mapped. *a*: mapped CML-specific strongly coexpressed pairs. *b*: mapped CML-specific weakly coexpressed pairs. *c*: mapped normal-specific strongly coexpressed pairs. *d*: mapped normal-specific weakly coexpressed pairs.

**Table 3:** Mapping coexpressed gene pairs to annotated gene pairs from each GO term for cellular component.

GO terms	Fisher exact test					Corrected *P* value
	*a *	*b *	*c *	*d *	*P* value	
**Ribonucleoprotein complex**	271	107	107	271	**<0.001**	**<0.018**
**Ribosome**	62	9	9	62	**<0.001**	**<0.018**
**Cytosolic ribosome**	22	4	4	22	**<0.001**	**<0.018**
**Ribosomal subunit**	26	4	4	26	**<0.001**	**<0.018**
**Cytosol**	273	127	127	273	**<0.001**	**<0.018**
**Cytosolic part**	34	8	8	34	**<0.001**	**<0.018**
Large ribosomal subunit	14	10	10	14	0.193	3.474
**Intracellular non-membrane-bounded organelle**	281	157	157	281	**<0.001**	**<0.018**
**Intracellular organelle part**	459	265	265	459	**<0.001**	**<0.018**
**Cytosolic large ribosomal subunit**	10	0	0	10	**<0.001**	**<0.018**
**Cytoplasmic part**	481	273	273	481	**<0.001**	**<0.018**
**Cytoplasm**	704	416	416	704	**<0.001**	**<0.018**
**Intracellular organelle**	819	413	413	819	**<0.001**	**<0.018**
**Nuclear part**	138	73	73	138	**<0.001**	**<0.018**
**Nuclear lumen**	103	57	57	103	**<0.001**	**<0.018**
**Intracellular organelle lumen**	123	80	80	123	**<0.001**	**<0.018**
Spliceosome	11	7	7	11	0.159	2.862
**Nucleolus**	41	19	19	41	**<0.001**	**<0.018**

GO: gene ontology. GO terms highlighted in bold text are significantly mapped. *a*: mapped CML-specific strongly coexpressed pairs. *b*: mapped CML-specific weakly coexpressed pairs. *c*: mapped normal-specific strongly coexpressed pairs. *d*: mapped normal-specific weakly coexpressed pairs.
