# Supplementary material for: Coexpression Pattern Analysis of NPM1-Associated Genes in Chronic Myelogenous Leukemia
Source: Biomed Res Int. 2015 Apr 15;2015:610595. doi: 10.1155/2015/610595 (PMC4413041; doi:10.1155/2015/610595)
Supplement: Supplementary file 2 [file 610595.f2.pdf]

Table S2. Biological process_Enriched GO terms for the functional annotation of NPM1-associated genes.

| # | Enriched GO terms | Genes found in our data | EASE score | FDR |
|---|---|---|---|---|
| 1 | Translational elongation | 35 | $6.80x10^{-51}$ | $9.00x10^{-48}$ |
| 2 | Translation | 38 | $3.20x10^{-36}$ | $4.20x10^{-33}$ |
| 3 | Cellular protein metabolic process | 53 | $6.00x10^{-18}$ | $7.90x10^{-15}$ |
| 4 | RNA processing | 23 | $8.60x10^{-12}$ | $1.10x10^{-08}$ |
| 5 | RNA metabolic process | 26 | $1.70x10^{-09}$ | $2.20x10^{-06}$ |
| 6 | mRNA processing | 14 | $2.40x10^{-07}$ | $3.20x10^{-04}$ |
| 7 | RNA splicing | 13 | $4.70x10^{-07}$ | $6.20x10^{-04}$ |
| 8 | mRNA metabolic process | 14 | $1.20x10^{-06}$ | $1.60x10^{-03}$ |

GO: gene ontology. EASE score: a modified Fisher exact test, Expression Analysis Systematic Explorer score. FDR: false discovery rate.

Table S3. Cellular component_Enriched GO terms for the functional annotation of NPM1-associated genes.

| # | Enriched GO terms | Genes found in our data | EASE score | FDR |
|---|---|---|---|---|
| 1 | Ribonucleoprotein complex | 51 | $6.40x10^{-49}$ | $7.60x10^{-46}$ |
| 2 | Ribosome | 35 | $1.80x10^{-39}$ | $2.10x10^{-36}$ |
| 3 | Cytosolic ribosome | 26 | $7.90x10^{-37}$ | $9.40x10^{-34}$ |
| 4 | Ribosomal subunit | 29 | $1.50x10^{-36}$ | $1.80x10^{-33}$ |
| 5 | Cytosol | 53 | $5.90x10^{-31}$ | $7.10x10^{-28}$ |
| 6 | Cytosolic part | 27 | $6.80x10^{-31}$ | $8.10x10^{-28}$ |
| 7 | Cytosolic small ribosomal subunit | 14 | $9.10x10^{-20}$ | $1.10x10^{-16}$ |
| 8 | Small ribosomal subunit | 15 | $1.40x10^{-18}$ | $1.60x10^{-15}$ |
| 9 | Large ribosomal subunit | 15 | $3.50x10^{-18}$ | $4.10x10^{-15}$ |
| 10 | Intracellular non-membrane-bounded organelle | 54 | $4.00x10^{-18}$ | $4.80x10^{-15}$ |
| 11 | Intracellular organelle part | 65 | $2.10x10^{-16}$ | $2.70x10^{-13}$ |

| | | | | |
|---|---|---|---|---|
| 12 | Cytosolic large ribosomal subunit | 12 | $3.10x10^{-16}$ | $4.00x10^{-13}$ |
| 13 | Cytoplasmic part | 67 | $2.20x10^{-14}$ | $2.60x10^{-11}$ |
| 14 | Cytoplasm | 79 | $5.90x10^{-13}$ | $7.00x10^{-10}$ |
| 15 | Intracellular organelle | 82 | $2.50x10^{-09}$ | $3.00x10^{-06}$ |
| 16 | Nuclear part | 33 | $2.00x10^{-08}$ | $2.30x10^{-05}$ |
| 17 | Nuclear lumen | 29 | $2.90x10^{-08}$ | $3.40x10^{-05}$ |
| 18 | Intracellular organelle lumen | 32 | $4.30x10^{-08}$ | $5.20x10^{-05}$ |
| 19 | Spliceosome | 10 | $1.30x10^{-07}$ | $1.50x10^{-04}$ |
| 20 | Nucleolus | 19 | $2.40x10^{-07}$ | $2.90x10^{-04}$ |
| 21 | Small nuclear ribonucleoprotein complex | 5 | $1.40x10^{-05}$ | $1.60x10^{-02}$ |

GO: gene ontology. EASE score: a modified Fisher exact test, Expression Analysis Systematic Explorer score. FDR: false discovery rate.
